# Epidemiological characteristics and distribution of congenital thumb duplication in south China: An analysis of 2,300 thumbs in 2,108 children

**DOI:** 10.3389/fped.2022.1027243

**Published:** 2022-11-02

**Authors:** JianPing Wu, WeiZhe Shi, XueMei Lin, JingChun Li, Zhe Yuan, Mingwei Zhu, YuanZhong Liu, YiQiang Li, Federico Canavese, HongWen Xu

**Affiliations:** ^1^ Department of Pediatric Orthopaedics, GuangZhou Women and Children’s Medical Center, GuangZhou, China; ^2^ Department of Pediatric Orthopedic Surgery, Lille University Center and Faculty of Medicine Henri Warenbourg, Jeanne de Flandre Hospital, Lille, France

**Keywords:** thumb duplication, epidemiology, classification, anatomy, morphology, associated anomaly

## Abstract

**Objective:**

The objective of this study was to evaluate epidemiological and anatomical characteristics of children with congenital thumb duplication (CTD).

**Methods:**

We retrospectively reviewed 2108 children with CTD. Data regarding sex, age at the surgery, laterality, uni- or bilateral involvement, and dominant side were retrieved from the medical charts. Plain radiographs were used to classify all CTD according to Wassel-Flatt, Rotterdam and Chung classification systems and to evaluate the patho-anatomy of the duplication as well as the presence of associated anomaly.

**Results:**

A total of 796 girls and 1,312 boys with CTD (*n* = 2,300 thumbs) met the inclusion criteria. The male to female and unilateral to bilateral ratio were 1.6:1 and 10:1, respectively. Associated anomaly was found in 238/2108 patients (11.3%), and the middle phalanx deformity of the 5th finger was the most common one. A dominant thumb, larger and more developed, was on the ulnar side in 2270/2,300 cases (98.7%).

According to the Wassel-Flatt classification, type IV (40.2%) was the most common deformity and the extra thumb was connected to the main thumb by a joint in most cases (437/780); overall, 15.7% of thumbs (*n* = 360) did not fit the Wassel-Flatt classification.

According to the Rotterdam classification, type IV (51.3%) was the most common form; in most cases (363/1180) the thumb was hypoplastic or floating. Overall, 3/2,300 thumbs (0.1%) could not be classified according to Rotterdam classification.

According to the Chung classification, type A was the most common subtype (44.1%); in most cases (716/1015) the duplication was at the level of the metacarpal bone. Overall, 2/2,300 thumbs (0.1%) did not fit the Chung classification.

**Conclusions:**

In patients from southern China, CTD shows male and right-sided predominance with ulnar-dominant thumb. Abnormalities of the middle phalanx of the 5th finger are more frequent in patients with associated anomaly. The development of a simple and comprehensive classification system is needed to guide treatment and to adequately assess the epidemiological characteristics of patients with CTD in order to facilitate comparison between different patients' populations.

**Level of evidence:**

III

## Introduction

The incidence of congenital duplication of the thumb (CTD) is particularly frequent in Americans, Japanese, and Chinese, and is estimated to range from 0.08‰ to 7.6‰; however, its etiology is still unknown (1–4).

CTD most commonly affects the right hand and may be associated with cardiac abnormalities and syndactylism. Based on the different patho-anatomy CTD deformity can be classified into 7 grades according to the system introduced by Wassel-Flatt (2). Despite the fact that several authors have reported that all forms of CTD can be classified according to the scheme proposed by Wassel-Flatt (2, 5–7), in practice, some types do not fit into this classification.

For example, Su et al. and Li et al. reported, respectively, that 14.5% and 17.8% of CTDs cannot be classified according to the Wassel-Flatt classification since this is based only on the level of thumb duplication and not on the morphological characteristics of the bone (8, 9). In fact, it therefore does not allow the development of a treatment algorithm. To overcome some of these problems, the Rotterdam classification by Zuidam et al. can identify all forms of CTD. However, it is relatively complex and difficult to use in clinical practice (10). In addition, this classification does not allow precise characterization of the accessory fingers, which may be connected to the main finger by joint, epiphysis, bone, or soft tissue, and these patho-anatomical features influence the choice of surgical treatment.

Similarly, the classification by Chung et al., based on the anatomy of the duplication, can classify all CTD and aid in the selection of the most appropriate surgical treatment; however, it does not include triphalangia and it does not precisely define the level of duplication (11).

Hu et al. compared the three classification systems and could not recommend one over the other (12). Several series have also investigated the epidemiological characteristics of CTD in different countries (3, 5–9, 13). However, no study to date has specifically evaluated the epidemiological and radiographic characteristics of CTD of children from south China using the three most common classification systems.

This article used the hand registry of Guangzhou Women and Children Medical Center (GWCMC) to examine the data of all children <15 years with CTD treated between 08/2015 and 04/2021. The objectives of this study were to investigate the epidemiological characteristics of CTD in children treated at GWCMC, and to evaluate, based on plain radiographs, the level of duplication and the morpho-anatomical characteristics of the duplication.

## Materials and methods

After securing Institutional Review Board (IRB) approval of Guangzhou Women and Children's Medical Centre and obtaining informed consent from the parents or legal guardians of the study participants prior to study commencement, we retrospectively reviewed the medical records of 2018 children who were diagnosed with CTD (*n* = 2300 thumbs), from 08/2015 to 04/2021, at our Institution.

The inclusion criteria were as follows: (1) confirmed diagnosis of CTD; (2) complete clinical and radiographic data; (3) treatment exclusively performed at our Institution. Patients with incomplete clinical and radiographic data and those managed elsewhere were excluded.

Data regarding sex, age at surgery, laterality, uni- or bilateral involvement, and dominant side were retrieved from the medical charts.

Plain radiographs were used to classify all CTD according to Wassel-Flatt, Rotterdam and Chung classification systems; plain radiographs were also used to evaluate the patho-anatomy of the duplication as well as the presence of associated anomaly.

This study received approval from the Institutional Review Board (IRB) of Guangzhou Women and Children's Medical Centre.

### Statistical analysis

All statistical analyses were performed using the statistics package SPSS 22.0 (SPSS, Chicago, IL, USA). Categorical parameters are expressed as frequencies and percentages. Quantitative data are expressed as the mean ± standard deviation and range.

## Results

The total number of patients admitted for CTD during study period was 2,190. Overall, 2,108 out of 2,190 (96.3%) patients (796 girls and 1,312 boys; 2,300 fingers) met the inclusion criteria; 65/82 were excluded due to incomplete data and 17/82 were excluded because they were treated in another institution. The average age at the time of surgery was 23.5 ± 1.5 months (range, 3–159).

Table 1 outline sex, laterality, uni- or bilateral involvement, dominant side according to the type of CTD as per Wassel-Flatt, Rotterdam and Chung classification systems (Table 1).

**Table 1:** Wassel-Flatt, Rotterdam and Chung subtypes according to demographic characteristics of patients.

Characteristics		Sex		Side				*Dominant thumb*	
		Male	Female	Left	Right	Unilateral	Bilateral	Ulnar	Radial
*Wassel*	**I**	0	4	2	2	0	4	4	0
	**II**	225	148	143	230	329	44	372	1
	**III**	54	32	31	55	69	17	83	3
	**IV**	453	327	326	454	682	98	773	7
	**V**	111	56	55	112	135	32	167	0
	**VI**	103	55	63	95	117	41	144	14
	**VII**	247	125	134	238	296	76	368	4
	**Floating thumb**	177	92	131	138	206	63	269	0
	**Particular morphology**	61	30	37	54	82	9	90	1
*Rotterdam*	**I**	0	4	2	2	0	4	4	0
	**II**	339	214	224	329	485	68	552	1
	**III**	59	37	32	56	62	34	92	4
	**IV**	725	455	700	480	999	181	1170	10
	**V**	152	76	46	182	150	78	228	0
	**VI**	153	83	104	132	170	66	221	15
	**No classification**	3	0	0	3	3	0	3	0
*Chung*	**A**	632	383	385	630	864	151	1005	10
	**B**	94	71	73	92	137	28	164	1
	**C**	211	117	114	214	259	69	324	4
	**D**	492	298	350	440	654	136	775	15
	**No classification**	2	0	0	2	2	0	2	0
	**Total**	1431	869	922	1378	1916	384	2270	30

Overall, the male-to-female ratio was 1.6:1. The right side was involved in 1,186 patients (56.3%) and the left in 730 (34.6%); bilateral involvement was observed in the remaining 192 patients (9.1%). The unilateral-to-bilateral ratio was 10:1. Among unilateral cases, 0.2% (3/1916) of duplications had the duplicated thumb larger than the contralateral thumb while 99.8% had the main duplicated thumb less developed and smaller than the contralateral thumb. Of the 2,300 fingers, the larger duplicated thumb was located on the ulnar side in 2,270 (98.7%) (Table 1).

**Figure 1:**
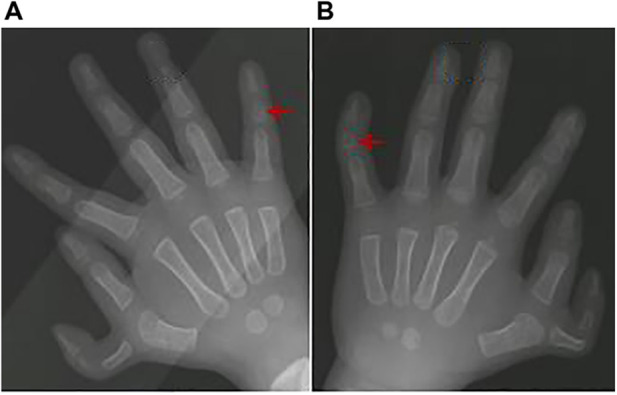
The most common associated anomaly: the red arrow highlights the middle phalanx anomalies of little finger.

The presence of associated anomaly was recorded in 238 patients (302 thumbs). The most common associated anomaly was at the middle phalanx of the 5th finger (197 patients, 222 thumbs) (Figure 1); the remaining 80 thumbs (51 patients) had limb abnormalities such as other forms polydactyly (11 patients, 16 thumbs) and syndactyly (5 patients, 7 thumbs), facial dimorphism (8 patients, 8 thumbs), congenital heart disease (6 patients, 8 thumbs), and other congenital anomalies such as scoliosis, ipsilateral thumb hypoplasia, imperforate anus, citrin deficiency and cryptorchidism or syndrome such as Down's syndrome (21 patients, 26 thumbs).

**Figure 2:**
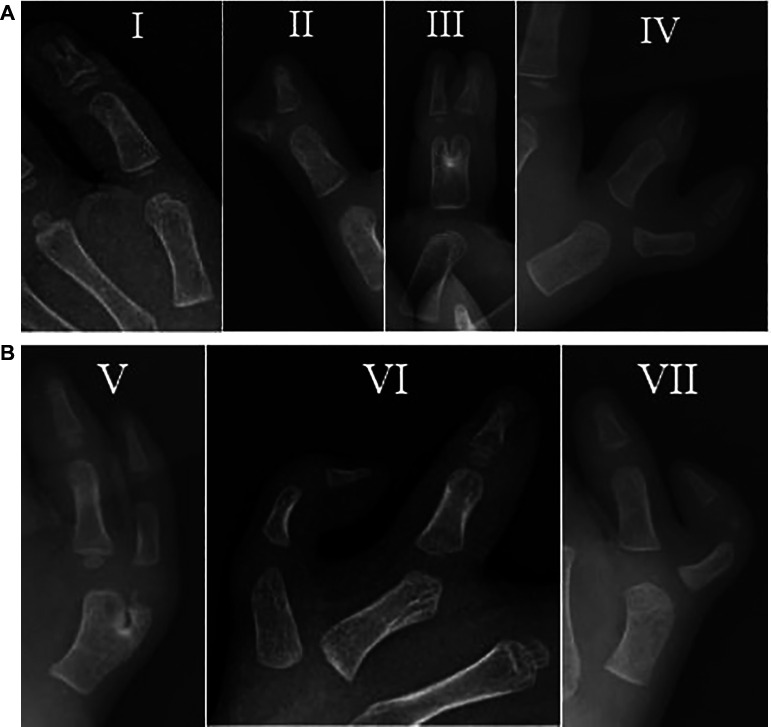
(**A,B)** Radiograph of the Wassel-Flatt classification: (**A**) type I, II, III and IV; (**B**) type V, VI and VII.

**Figure 3:**
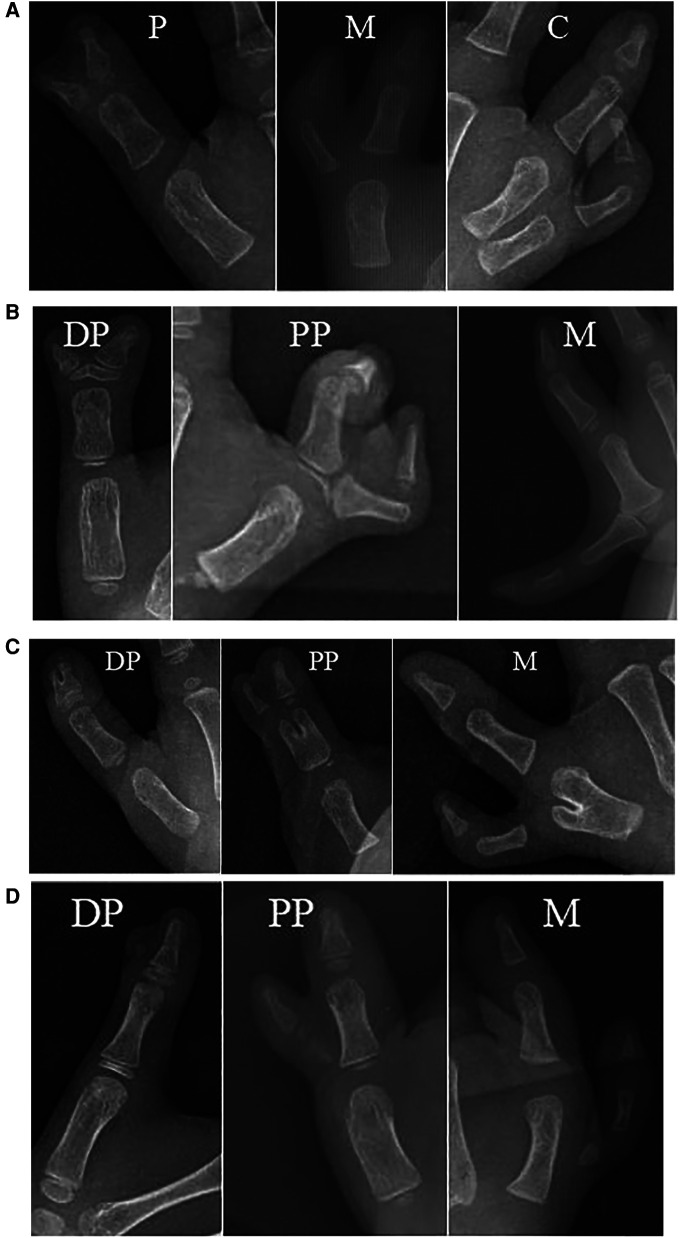
(**A–D)** Radiograph of the Chung et al. Classification. (**A**) type A-P, A-M, A-C; (**B**) type B-DP, B-PP and B-M; (**C**) type C-DP, C-PP and C-M; (**D**) type D-DP, D-PP and D-M. P, phalanx; M, metacarpal; C, carpal; DP, distal phalanx; PP, proximal phalanx.

**Figure 4:**
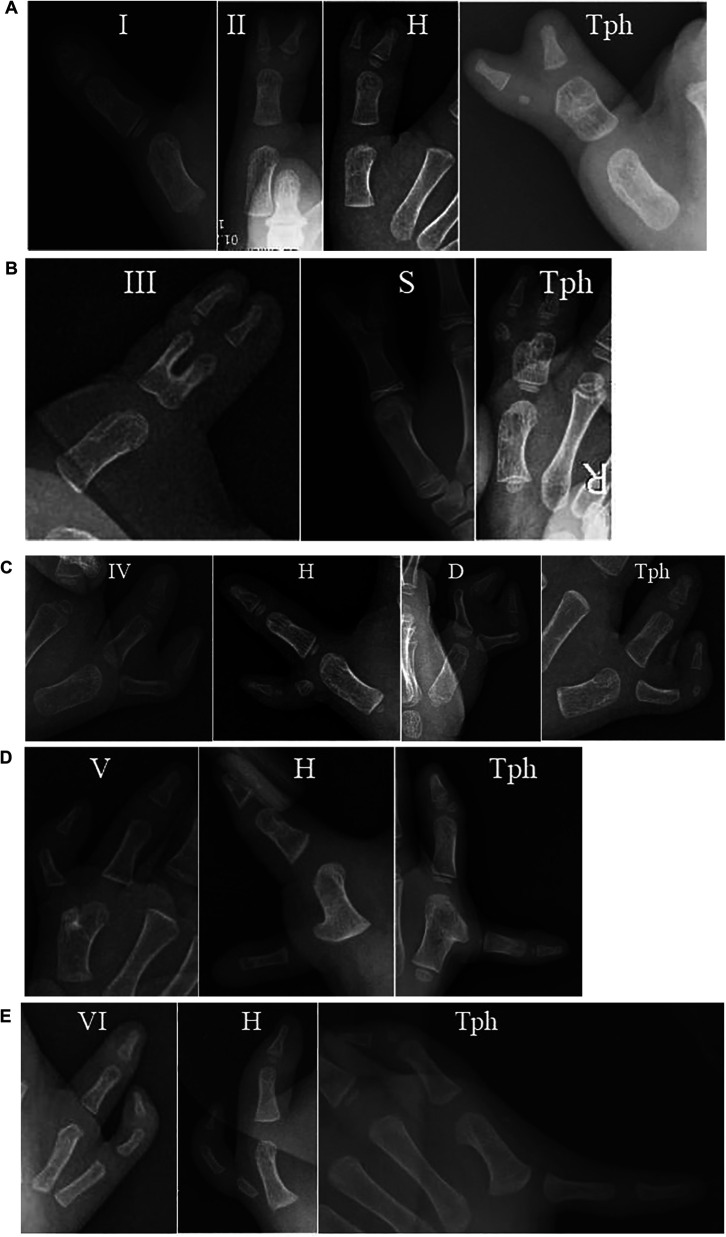
(**A–E)** Radiograph of the Rotterdam Classification. (**A**) type I, II-II, II-H and II-Tph; (**B**) type III-III, III-S and III-Tph; (**C**) type IV-IV, IV-H, IV-D and IV-Tph; (**D**) type V-V, V-H and V-Tph; (**E**) type VI-VI, VI-H and VI-Tph. H, hypoplastic or floating; D, deviation; Tph, triphalangism; S, symphalangism.

Table 2 summarizes the anatomical characteristics of CTD and the different duplication level according to the three classification systems (Figures 2–4).

**Table 2:** Comparison of the proportion of the anatomical characteristics of the extra thumb connected to the main thumb in patients with CTD.

Anatomical characteristics			Joint	Bone	Soft tissue	Triphalangia	Total
*Wassel*	I		–	4	–	–	4
	II		238	14	121	–	373
	III		–	86	–	–	86
	IV		437	83	260	–	780
	V		–	167	–	–	167
	VI		49	28	81	–	158
	VII		286	65	21	372	372
*Rotterdam*	I		–	4	–	–	4
	II	II	261	–	–	5	261
		H	1	11	275		287
		Tph	5	–	–		5
	III	III	–	86	–	9	86
		S	–	1	–		1
		Tph	–	9	–		9
	IV	IV	429	–	–	303	429
		H	3	18	342		363
		D	7	78	–		85
		Tph	282	3	18		303
	V	V	–	167	–	60	167
		H	–	1	–		1
		Tph	–	60	–		60
	VI	VI	77	–	–	6	77
		H	1	2	150		153
		Tph	–	2	4		6
*Chung*	A	*P*	247	–	–	8	247
		M	716	–	–	278	716
		C	52	–	–	1	52
	B	DP	–	28	–	–	28
		PP	–	104	–	2	104
		M	–	33	–	4	33
	C	DP	–	4	–	–	4
		PP	–	93	–	9	93
		M	–	231	–	60	231
	D	DP	–	–	170	–	170
		PP	–	–	315	7	315
		M	–	–	305	15	305

H, hypoplastic or floating; D, deviation; Tph, triphalangism; S, symphalangism; P, phalanx; M, metacarpal; C, carpal; DP, distal phalanx; PP, proximal phalanx.

## Discussion

The present study from a large cohort of patients from south China found CTD is more frequent in boys, is often unilateral, and frequently affects the right side. In addition, the ulnar thumb usually develops more than the radial one and middle phalanx anomalies of 5th finger are particularly frequent in patients with associated anomaly. The Wassel-Flatt classification is not comprehensive. Although the Rotterdam and Chung classification systems are more comprehensive, they have limitations that preclude their use in clinical and surgical practice (8–10, 12).

Our findings showed CTD is highly prevalent in boys, which is in accordance with most previous reports. In particular, Lin et al., Su et al., Li et al. and Yen et al. reported a male to female ratio ranging between 1.7/2 and 1 while the ratio in our series ranged between 1.6 and 1 (3, 8, 9, 14). However, Ozalp et al. and Islam et al. reported a male to female ratio of 1:1 and a slightly larger proportion of females in some regions of the Middle East and Europe as reported by Cabrera-González et al., Al-Qattan et al., and Manske et al. (6, 15–18). These discrepancies may be related to differences in ethnicity, samples, economic status, education, medical level, environment and diet (3, 8, 9, 14).

Previous studies have pointed out unilateral cases are more common than bilateral cases in patients of different ethnicity (3, 8, 9, 16–19). In unilateral cases, the right hand is more likely to be affected in reports from Japan, Spain, Turkey and China, which is consistent with our findings (3, 8, 9, 16, 18, 19). In contrast, a slightly left-sided (1.05) trend was reported in Arabia by Al-Qattan et al. (16).

In this study, we found that most (99.9%) duplicated thumbs were less developed than the normal side, which was in accordance to the work by Lin et al. Wang et al. reported that in patients with CTD, a permanent developmental stability exists between the thumb and the index finger and the development of the thumb is not influenced by the ablation of the duplicated thumb (3, 20). They recommended that the size of the main thumb should require augmentation by soft tissue reconstruction or Bilhaut-Cloquet procedures if the postoperative thumb is less than 80% of the opposite one.

We found that most of (98.7%) the larger duplicated thumbs were located on the ulnar side, which is also consistent with previous reports (1, 3, 14, 21, 22). Moreover, our findings showed that the ulnar thumb was less commonly associated with clinodactyly than the radial thumb, which might be used to guide surgical approaches and procedures in agreement with Lin et al. (3).

In our group, 238 cases had associated anomalies, a rate (11.3%) lower than that reported by Chung et al. (31.7%) and Cabrera-González et al. (17%), and higher than that reported by Lin et al. (6.1%) (3, 11, 16). The most common associated anomaly was a deformity at the level of the middle phalanx of the 5th finger (82.8%), which was not reported in previous studies and it was inconsistent with Chung el al. (syndactyly, 64.4%) and Lin et al. (congenital heart disease, 19.2%). In contrast, syndactyly and congenital heart disease were present in 2.1% and 2.5%, respectively (3, 11, 16). Table 3 shows other forms polydactyly, syndactyly, congenital heart disease and ipsilateral thumb hypoplasia are common in patients with CTD (3, 11, 16, 23). Other forms polydactyly, syndactyly and congenital heart disease were most common in patients with Wassel-Flatt type II and IV, type IV and type VI, respectively. There was only one patient was affected by Down syndrome which was consistent with Lin et al. but not Cabrera-Gonzalez et al. which was Treacher Collins syndrome. Moreover, there was one patient with Wassel-Flatt type VII had ipsilateral thumb hypoplasia also reported by Lin et al. (2 cases) and Bauer et al. (10 cases with Wassel-Flatt type IV and higher) (Table 3).

**Table 3:** Previous studies about association between congenital anomalies and CTD.

Associated anomalies (*n*)	Most common (*n*/%)	Other forms polydactyly (*n*/%)	Syndactyly (*n*)	Congenital heart disease (*n*/%)	Ipsilateral thumb hypoplasia (*n*/%)	Syndrome (*n*/%)	Others
Wu JP this study *n* = 238/2108	The middle phalanx of the 5th finger 197/82.8	11/4.6	7/2.9	6/2.5	1/0.4	Down syndrome 1/0.4	Citrin deficiency Scoliosis Imperforate anus Cryptorchidism et al.
Chung et al. (3) *n* = 45/159	Syndactyly 29/64.4	–	29/64.4	6/13.3	–	–	Clinodactyly Triphalangeal thumb et al.
Lin et al. (11) *n* = 26/428	Congenital heart disease 5/19.2	5/19.2	3/11.5	5/19.2	2/7.7	Down syndrome 1/3.8	G6PD deficiency Ear abnormality Scoliosis et al
Bauer et al. (23) *n* = 10/132	Ipsilateral thumb hypoplasia 10/8.2	–	–	–	10	–	–
Cabrera-Gonzalez et al. (16) *n* = 17/99	–	–	–	–	–	Treacher Collins syndrome 1/5.9	–

G6PD, glucose-6-phosphate dehydrogenase.

Overall, 15.7% of CTD could not be classified according to the Wassel-Flatt classification, which was similar to Li et al. (*n* = 2562) and Su et al. (*n* = 325) that found 17.8% and 14.5% of cases could not be classified according to the Wassel-Flatt classification, respectively (8, 9). Although the Wassel-Flatt system is simple and practical, it does not include several varieties of polydactyly (24). In addition, it is mainly based on the level of duplication and not on the anatomical characteristics. For example, in type II and VII, the extra thumb can be connected to the main thumb by bone, joint or soft tissue (Table 2).

The Rotterdam classification has broader classification possibilities than the Wassel-Flatt classification, but it is more complex and it is difficult to use in clinical practice (10). For example, in type IV, the extra thumb also can be connected to the main thumb by bone, joint or only soft tissue, it can be hypoplastic or floating, and it can present triphalangia or axial deviation. The classification by Chung et al. includes 4 types of CTDs based on the anatomy of the duplication although it does not include triphalangia nor precisely define the level of the duplication (11). For example, in type A, the extra thumb is connected to the main thumb by a joint at the level of the phalanx, metacarpal and carpal bones (Table 2).

The choice of treatment varies depending on the anatomy of the extra thumb connected to the main thumb, which is consistent with the view of previous reports (10, 11, 24–27). Tada et al. and Kim et al. reported that an extra thumb connected to the main thumb by soft tissue alone should be classified as an independent subtype (24, 25). For example, in C type, the extra thumb can be connected to the main thumb by bifurcated phalanx at the level of the distal phalanx, proximal phalanx or bifurcated metacarpus. Flap design, muscle tendon and bone reconstruction are different depending on the duplication level (27, 28). Therefore, it is necessary to explore a simple and comprehensive classification system useful for selecting the best treatment option (29).

We encountered some limitations in the analysis of our results. This is a retrospective review. However, it includes a large cohort of patients and all subtypes of CTD are likely to be represented. Patienst are from south China, so it is possible that some differences may exist among countries. The data from the present study may not be universally accepted although they can be used for comparison with other Asian and not-Asian countries. Our patients did not undergo genetic testing so it is not possible to evaluate the rate and type of genetic disorder associated with CTD. We assessed our results with three classification systems, but each one has its own limitations. There is space for developing new classifications systems, fully comprehensive and easy to use in clinical practice, in order to be more precise when performing epidemiological analysis.

## Conclusion

In patients from southern China, CTD shows male and right-sided predominance with ulnar-dominant thumb. Abnormalities of the middle phalanx of the 5th finger are more frequent in patients with associated anomaly. The development of a comprehensive classification system is needed to adequately assess the epidemiological and the anatomical characteristics of patients with CTD in order to facilitate comparison between different patients' populations and to guide treatment.

## Data Availability

The raw data supporting the conclusions of this article will be made available by the authors, without undue reservation.
